# Injury Profiles of Police Recruits Undergoing Basic Physical Training: A Prospective Cohort Study

**DOI:** 10.1007/s10926-022-10059-2

**Published:** 2022-08-02

**Authors:** Nicole Merrick, Nicolas H. Hart, Andrea B. Mosler, Garth Allen, Myles C. Murphy

**Affiliations:** 1 School of Medical and Health Sciences, Edith Cowan University, Joondalup, Western Australia Australia; 2 Caring Futures Institute, College of Nursing and Health Science, Flinders University, Adelaide, SA Australia; 3 Institute for Health Research, The University of Notre Dame Australia, Fremantle, Western Australia Australia; 4 La Trobe Sport and Exercise Medicine Research Centre, La Trobe University, Bundoora, Victoria Australia; 5 Western Australian Police Academy, Western Australian Police Force, Joondalup, Western Australia Australia; 6 Nutrition and Health Innovation Research Institute, School of Medical and Health Sciences, Edith Cowan University, Joondalup, Western Australia Australia; 7 School of Health Sciences and Physiotherapy, The University of Notre Dame Australia, Fremantle, Western Australia Australia

**Keywords:** Law enforcement, Injury surveillance, Injury epidemiology, Injury burden

## Abstract

*Purpose* A lack of published epidemiological data among police recruits presents a major challenge when designing appropriate prevention programs to reduce injury burden. We aimed to report the injury epidemiology of Western Australian (WA) Police Force recruits and examine sex and age as injury risk factors. *Methods* Retrospective analyses were conducted of prospectively collected injury data from WA Police Force recruits between 2018–2021. Injury was defined as ‘time-loss’ and injury incidence rate per 1000 training days (Poisson exact 95% confidence intervals) was calculated. For each region and type of injury, the incidence, severity, and burden were calculated. The association between age, sex, and injury occurrence were assessed using Cox regression time-to-event analysis. *Results* A total of 1316 WA Police Force recruits were included, of whom 264 recruits sustained 304 injuries. Injury prevalence was 20.1% and the incidence rate was 2.00 (95%CI 1.78–2.24) injuries per 1000 training days. Lower limb injuries accounted for most of the injury burden. Ligament/ joint injuries had the highest injury tissue/pathology burden. The most common activity injuring recruits was physical training (31.8% of all injuries). Older age (Hazard Ratio = 1.5, 95%CI = 1.2 to 1.9, *p* = 0.002) and female sex (Hazard Ratio = 1.4, 95%CI = 1.3 to 1.6, *p* < 0.001) increased risk of injury. *Conclusion* Prevention programs targeting muscle/tendon and ligament/joint injuries to the lower limb and shoulder should be prioritised to reduce the WA Police Force injury burden. Injury prevention programs should also prioritise recruits who are over 30 years of age or of female sex, given they are a higher risk population.

**Supplementary Information:**

The online version contains supplementary material available at 10.1007/s10926-022-10059-2.

## Introduction

Law enforcement officers perform vigorous physical training as a part of their recruit training program [1], similarly to military recruits [2]. These programs are designed to ensure that the recruit, upon graduation and deployment, is physically capable of performing the more strenuous components of their role, given the propensity of the occupation to physical injury [3, 4]. Physical fitness and resilience is therefore essential for law enforcement officers as their physical work demands are far higher than the general population [5]. It has also been identified that quantifying injury risk for qualified law enforcement officers is important to further reduce injury risk [6]. However, as with all intense physical training programs, injury and illness are common adverse events for law enforcement recruits, a population currently under-reported within the law enforcement and epidemiological literature [1, 7–9].

Our recent systematic review of law enforcement recruits (*n* = 6 police recruits, *n* = 1 border police recruits, *n* = 1 Federal Bureau of Investigation (FBI) recruits) identified a sparsity of data in relation to the injury severity and burden experienced by police recruits [10]. Specifically, no studies reported injury burden, and the methods of reporting injury region, type and activity of injury varied, significantly complicating the synthesis of data from different studies [10]. Furthermore, conflicting results were reported between studies concerning the frequency of injuries sustained between body regions [10]. Limited data from relatively small studies and mostly of low methodological quality means the certainty of the existing body of literature on injury epidemiology for law enforcement recruits is very low [10].

Knowledge of injury epidemiology forms an essential component of developing an injury prevention strategy [11]. Due to a lack of existing data on injury epidemiology in police recruits, designing a prevention program to specifically and effectively reduce injury burden in this population is significantly hampered. Using an example from elite football, ankle joint injuries are more common than quadriceps muscle injuries, yet quadriceps muscle injuries result in players missing more time from training and matches [12]. Thus, in this example, quadriceps muscle injuries have a larger *injury burden* than ankle injuries in elite football, even though they are less common overall. Accordingly, injury prevention programs targeting quadriceps muscle injury prevention would be more likely to increase overall player availability. Due to limitations of previous studies [10], we do not know what the burden of different injuries are within police recruits. Thus, current injury prevention programs based on injury frequency, may be ineffective and wasteful for the recruits and the law enforcement agency.

Influences of age and sex on injury incidence with law enforcement officers is also unknown. One study of police officers demonstrated females are at higher risk for moderate general health concerns [13] with another demonstrating higher levels of stress in female officers [14]. Additionally, one small study of FBI recruits reported older age as a risk factor for injury [15]. These findings support quantifying the risk associated with sex and age of recruits undergoing basic training. In sporting populations, age [16] and sex [17] influence injury incidence; which has also been observed in military populations, undergoing basic training, for age [18] and sex [19]. Knowledge of these key influences are important when developing physical training and injury prevention programs. However, due to the dearth of studies in law enforcement recruits exploring these risk factors, it is unknown whether age and sex are risk factors in a police cohort.

The aim of this study was to examine and report the injury epidemiology of Western Australian (WA) Police Force recruits undergoing basic physical training using prospectively collected injury data.

### Objectives

1. Report the frequency, prevalence, incidence rate, severity, and burden of injury in WA Police force recruits undergoing physical training.
2. Examine the association between recruit age and sex and injury occurrence.

## Methods

### Study Design

A retrospective analysis was conducted of prospectively recorded injury data from WA Police Force recruits collected from 2018 to 2021.

### Ethical Approvals

This project was approved by the Edith Cowan University Human Research Ethics Committee (HREC ID: 2021-02982-MURPHY) and WA Police Force Research Governance division.

### Data Availability

Data from this study will not be freely available online. However, the authors, in consultation with the WA Police Force Research Governance division, will consider requests to access these data in the interests of research transparency and data synthesis (e.g., the data were required for an individual patient data meta-analysis) [20].

### Setting

Applicants seeking to join the WA Police Force are initially pre-screened using a battery of physical, intellectual, and psychological tests constituting the current WA Police Force selection process. Once potential applicants meet the selection criteria, they are inducted into the WA Police Force Academy as a recruit. Recruits then undergo vigorous physical training, including operational skills training (e.g., learning how to discharge firearms) and empty hand training (e.g., hand-to-hand combat). WA Police Force recruit training lasts for six-months before recruits are graduated and operationally deployed as a police officer.

Recruits who sustain injuries during their academy training program are reviewed by a network of general practitioners and physiotherapists and provided the recommended care. These clinicians complete an injury record form that is provided to the WA Police Force and entered into the WA Police Force recruit injury database. This database records the injured recruit’s squad, surname, injury diagnosis (e.g., Achilles tendinopathy), days into training the injury occurred, and the number of days the recruit required training to be modified due to injury. A separate physical performance database contains more detailed demographic data on all recruits who undergo recruit training, including the recruit’s squad, surname, sex and age (recorded within the database as 18–29 years or ≥ 30 years old).

### Injury Definition and Categorization

Due to the athletic nature of the training performed by Police Force recruits, and that prevention interventions will likely mirror those performed in athletic populations, in consultation with WA Police Force representatives, it was decided that the most informative categorisation of injury region/ type and injury tissue/ pathology would be those recommended for athletic populations. Therefore, this study used the recommended terminology and methods reported within the International Olympic Committee consensus statement on methods for recording and reporting epidemiological data related to sport and physical activity injuries [21]. This was important as previous studies have not differentiated or defined key injury terminology, such as the injury classification (medical attention versus time loss), incidence, burden etc. A complete list of operational definitions used for study are presented in Table 1.

**Table 1:** Operational definitions

Exposure day	One day of WA Police force recruit training represents one exposure day
Injury	Injury occurring during the six-month WA Police force recruit training program that required modification from physical training following consultation with a medical practitioner or physiotherapist
Injury region/ area	Body region/area as per the International Olympic Committee recommendations (e.g., ankle or shoulder) [21]
Injury tissue/ pathology type	Tissue/pathology type as per the International Olympic Committee recommendations (e.g., muscle/tendon or ligament/joint capsule). [21]
Activity of injury	The activity the recruit was performing that resulted in sustaining an injury (e.g., physical training versus empty hand training)
Injury frequency	The number of injuries sustained (n)
Injury prevalence	Number of WA Police force recruits injured compared to all recruits participating in the six-month training program (%)
Injury incidence rate	Number of injuries per 1000 exposure days ((Σ injuries/Σ exposure days) × 1000)
Injury severity	Number of days requiring modification from WA Police force recruit training
Very mild injury severity	0–3 days training modification
Mild injury severity	4–7 days training modification
Moderate injury severity	8–28 days training modified
Severe injury severity	More than 28 days training modified
Injury burden	Number of days modified per 1000 exposure days ((Σ days’ modified/Σ exposure days) × 1000)

Injuries were defined as requiring modification from physical training following consultation with a medical practitioner or physiotherapist (time-loss injury definition). Data from the WA Police Force recruit injury database and the WA Police Force physical performance database was linked to create a single dataset. Data linkage was performed by a single study author (NM) and compiled within a single Microsoft® Excel® for Microsoft 365 MSO (Version 2201) spreadsheet. The process of coding the injury diagnoses to a specific region and type was piloted by two members of the research team (NM and MM) who are clinical physiotherapists with experience diagnosing musculoskeletal injuries. This process involved assigning an injury body region or area and tissue or pathology type to the diagnosis provided within the WA Police Force recruit injury database. As an example, a diagnosis of a partial tear of the anterior talofibular ligament was recorded within our linked datasheet with the body region area being ankle and the tissue/pathology type being ligament/joint capsule.

### Exposure

Where a recruit completed the six-month WA Police Force recruit training program, they were assigned an exposure of 130 training days. Where a recruit was injured during the training program, they were assigned an exposure equal to that of the number of days into training the injury occurred.

### Duration of Injury and Days Into Training

In most instances, the duration of injury was reported in the WA Police Force recruit injury database. Where possible, when the duration of injury was missing, it was manually calculated using the injury date and return to physical training date recorded within the WA Police force recruit injury database.

### Missing Data

Where recruit age and sex were not available from the WA Police Force physical performance database, these were requested from the WA Police Force. Where these data were still unable to be provided, they were entered as ‘not noted’. Where no details were provided to allocate a body region to an injury, these entries were classified as ‘unspecified’. Where no details were provided to allocate a tissue/pathology type of injury they were entered as ‘non-specific’.

Where detail existed that allowed allocation of injury region and tissue/pathology type using clinical judgement, yet the diagnosis did not immediately allow allocation of injury region and tissue/pathology type, clinical judgement was used. For example, an entry of “abdominal pain” with a note of “GP prescribed antibiotics,” was entered as “infection” as the tissue/pathology type, with the prescription of antibiotics is highly suggestive of that. Furthermore, Where the body region was simply described as “back” the region was entered as “lumbosacral” unless notes suggested otherwise.

### Data Analysis

Data analysis was conducted using IBM SPSS Statistics (version 28.0.1.0). Descriptive statistics were used to report the available demographic data of the WA Police Force recruits. For injury, the area/region and tissue/pathology type, the frequency, severity, total time-loss and burden are presented (formulae for calculation are included within Table 1). The frequency of activity of injury for injured recruits is reported. The severity of injury was also reported as the frequency of very mild, mild, moderate, and severe injuries. Overall injury frequency, prevalence, and incidence rate (Exact Poisson 95% confidence intervals) were also reported.

Cox regression time-to-event (survival) analysis examined injury risk over time, when accounting for sex, age and year, reporting Hazard Ratio (HR) and 95% CIs, and the requirements for performing Cox regression time-to-event analysis were met. Data differentiating subsequent injury versus recurrent injury were not recorded by the WA Police Force. Therefore, due to established differences in the aetiology of recurrent versus subsequent injuries, [22, 23] we opted to not include these injuries within our statistical model. Cases with missing demographic data were excluded. Statistical significance was set at *p* < 0.05.

## Results

### Participants

A total of 1326 WA Police force recruits completed their basic training between 2018 and 2021. Ten recruits had injuries upon commencing at the WA Police Force Academy and were excluded from the cohort. Therefore, 1316 recruits were included within this study. Of the 1316 recruit records, 134 records did not report age or sex (10.2%). Age was stratified as 18–29 years or ≥ 30 years. Of the 1182 complete records, 725 (61.3%) recruits were aged between 18–29 years and 457 (38.7%) were aged ≥ 30 years. Of the 1182 complete records, 819 (69.3%) recruits were men, and 363 (30.7%) recruits were women. Additional demographic information, such as ethnicity, height or weight were not available.

### Exposure

There were 1052 recruits (79.9%) who did not sustain an injury and completed the six-month WA Police Force program, resulting in a total exposure of 136,760 training days. The injured sample (*n* = 264; 20.1%) recorded a total exposure of 15,318 training days. Thus, total exposure for WA Police Force recruits was 152,078 training days.

### Injuries

Between 2018 and 2021, 304 injuries were recorded by the WA Police Force. Of these 304 injuries, 264 were index injuries.

#### Injury Prevalence

A total of 264 WA Police recruits sustained at least one injury during the observation period (Table 2), resulting in a prevalence of 20.1% of recruits being injured during academy training. There were 1052 recruits who did not report an injury (Table 2).

**Table 2:** Number of injured recruits per year

Year	Injured, n (%)	Uninjured, n (%)	Overall, n (%)
2018	24 (9.1)	122 (11.6)	146 (11.1)
2019	71 (26.9)	234 (22.2)	305 (23.2)
2020	71 (26.9)	287 (27.3)	358 (27.1)
2021	98 (37.1)	409 (38.9)	507 (38.5)
Overall	264 (100)	1052 (100)	1316 (100)

*n* = number, % = percentage.

#### Injury Incidence Rate

The injury incidence rate was 2.00 injuries per 1000 training days (Poisson 95%CI 1.78–2.24 injuries per 1000 training days).

#### Injury Severity

The severity of injuries are presented within Table 3. Most injuries (84.2%) were classified as moderate or severe.

**Table 3:** The overall severity of injuries

Injury severity	Frequency, *n* (%)*
Very mild, 0–3 days training modified	4 (1.3)
Mild, 4–7 days training modified	28 (9.2)
Moderate, 8–28 days training modified	158 (52.0)
Severe, more than 28 days training modified	98 (32.2)

*n* = number, % = percentage

*16 records did not have severity of injury recorded.

### Injury Area/Region

The frequency, severity and burden by injury area/region is presented in Table 4 with further graphical representation of the burden presented in Online Resource A. The most common regions of injury were in the lower limb, accounting for 64.9% of all injuries (Table 4). However, shoulder injuries made up the second-highest injury burden, approaching 8 time-loss days per 1000 recruit training days.

**Table 4:** The frequency, severity and burden by injury region

Injury region	Frequency, *n* (%)	Injury incidence rate*	Injury severity^a^, M (SD)	Sum of days lost	Injury Burden*
Foot	19 (6.3)	0.12	30 (34)	575	3.78
Ankle	33 (10.9)	0.22	29 (23)	949	6.24
Lower leg	42 (13.8)	0.28	25 (15)	982	6.46
Knee	53 (17.4)	0.35	38 (48)	1794	11.80
Thigh	37 (12.2)	0.24	24 (27)	869	5.71
Hip/ groin	13 (4.3)	0.09	42 (36)	509	3.35
Lumbosacral	19 (6.3)	0.12	16 (11)	289	1.90
Thoracic spine	1 (0.3)	0.01	9 (N/A)	9	0.06
Trunk (abdomen)	6 (2.0)	0.04	22 (10)	130	0.85
Trunk (chest)	12 (3.9)	0.08	39 (29)	425	2.79
Neck	2 (0.7)	0.01	16 (13)	32	0.21
Hand	9 (3.0)	0.06	45 (36)	406	2.67
Wrist	8 (2.6)	0.05	38 (30)	265	1.74
Elbow	2 (0.7)	0.01	18.0 (0)	36	0.24
Upper arm	3 (1.0)	0.02	21 (8)	63	0.41
Shoulder	31 (10.2)	0.20	42 (41)	1210	7.96
Head	2 (0.7)	0.01	45 (N/A)	45	0.30
Multiple sites	3 (1)	0.02	39 (43)	118	0.78
Respiratory	6 (2.0)	0.04	17 (5)	100	0.66
Unspecified	3 (1.0)	0.02	55 (51)	166	1.09

* = Per 1000 training days

^a^injury severity = training days modified due to injury, *n* = number, % = percentage, M = mean, SD = standard deviation, N/A = SD could not be calculated as only a single injury recorded.

### Injury Tissue/Pathology Type

The frequency, severity and burden by injury type is presented within Table 5 with further graphical representation of the burden presented in Online Resource A. The most common types of injury were muscle/tendon (31.3% of all injuries) or ligament/joint capsule injuries (18.8% of all injuries).

**Table 5:** The frequency, severity and burden by injury type

Injury type	Frequency, *n* (%)	Injury incidence rate*	Injury severity^a^ M (SD)	Sum of days lost	Injury Burden*
Bone (stress injury)	4 (1.3)	0.03	55 (43)	219	1.44
Bone (contusion)	10 (3.3)	0.07	36 (34)	254	1.67
Bone (fracture)	8 (2.6)	0.05	67 (21)	535	3.52
Cartilage, synovium or bursa	23 (7.6)	0.15	36 (33)	832	5.47
Infection	7 (2.3)	0.05	15 (6)	106	0.70
Ligament or joint capsule	57 (18.8)	0.37	44 (48)	2448	16.10
Muscle or tendon	95 (31.3)	0.62	24 (21)	2244	14.76
Nervous system or brain	1 (0.3)	0.01	45 (N/A)	45	0.30
Superficial tissue (skin)	9 (2.9)	0.06	11 (3)	86	0.57
Non-specific	81 (26.6)	0.53	27 (27)	2001	13.16
Not reported	9 (3.0)	0.06	29 (25)	202	1.33

* = Per 1000 training days

^a^Injury severity = training days modified due to injury, *n* = number, % = percentage, M = mean, SD = standard deviation, N/A = SD could not be calculated as only a single injury recorded.

### Activity of Injury

The activities participated when injured are detailed within Online resource B. The most common activity causing recruit injury was physical training (31.8% of all injuries) followed by injuries in the recruit’s own time (19.7% of all injuries).

### Association Between Age, Sex, and Training Year and Injury

Cases where demographic data were absent were excluded from Cox time-to-event analysis. Our time-to-event analysis determined that female sex (HR = 1.4, 95%CI = 1.3 to 1.6, *p* < 0.001) and being 30 years or older (HR = 1.5, 95%CI = 1.2 to 1.9, *p* = 0.002) significantly increased the injury rate. There was no influence of training year on injury. The influence of age, sex and training year on injury occurrence is presented within Online resource C, alongside the Kaplan–Meier Curve.

## Discussion

Injury epidemiology in law enforcement recruits is poorly reported [10]. Our cohort represents the first study to clearly report the prevalence, incidence rate, severity and burden of injuries to police recruits. Furthermore, this is also the first study to report the injury region, injury type and activity of injury in police recruits using recommended definitions and terminology for physical activity injuries [21].

Our study demonstrated that one-fifth of WA Police Force recruits are injured during their recruit training program, comparable to data reported in other law enforcement studies [7–9, 24], and studies of Australian basic military training [25, 26]. The injury incidence rate in our study was 2.00 injuries per 1000 training days, which sits at the lower end of those reported within other law enforcement cohorts [1, 7–9, 15]. In our recent systematic review [10], we reported injury incidence rates of between 1.67 and 4.24 injuries per 1000 training days. The high level of heterogeneity between studies in the recruit demographics and training procedures captured in our review is likely to account for the wide range of injury incidence rates recorded. This limits comparability of these results with our study and emphasises the need to use standardised terminology, recording and reporting methods for injury surveillance.

Our research is the first to report the severity and burden of injuries to police recruits. Lower limb injuries made up most of the overall injury burden and is comparable to military basic training [27, 28]. However, shoulder injuries were the second highest injury area/region as far as injury burden in our police recruit cohort, suggesting prevention strategies for shoulder injuries also be prioritised. Muscle/tendon and ligament/joint capsule injuries made up most of the overall injury burden. Conversely, in military basic training, bone stress injuries are a substantial cause of injury burden [27, 28], however in our study this only represents a very small proportion of the injuries (1.3%), which is very likely due to differences in basic training program requirements and structure between the professions. The common injury tissue/pathology types presented within our study are more comparable to those of football players [29].

These data suggest that injury prevention interventions would be most appropriately targeted at lower limb and shoulder muscle/tendon and ligament/joint injuries to reduce the overall injury burden in WA Police Force recruits. Furthermore, 31.8% of injuries occurred due to physical training, which suggests that targeting injury prevention strategies towards physical training components (e.g., running, strength, etc.) is likely to result in the largest reduction in injury burden. However, as mentioned previously, the inclusion of shoulder specific prevention strategies would also be warranted given the high burden.

Our study also determined that older recruits (greater than 30 years of age), and female recruits were at significantly higher risk of injury, with age and sex as notable risk factors. Our findings mirror those seen within qualified Police officers and other law enforcement training programs, such as the FBI in the United States of America [13, 15]. This has important ramifications for the training and management of recruits within the WA Police Force and wider law enforcement community. These findings suggest that specifically targeting injury prevention programs at female recruits and recruits over 30 years of age is likely to be provide a meaningful reduction in injuries to law enforcement recruits.

While establishing injury epidemiology remains the first step in the planning of targeted injury prevention strategies, before successful interventions can be implemented, aetiology needs to be established [11]. Few studies have explored the risk factors that predispose law enforcement recruits to injury during their training block. Vertical jump height has been associated with injury (r = -0.09, *p* < 0.005) and explained 0.8% of the variance in injury rates (*p* < 0.005) [7]. Reduced left hand, not right hand, grip strength was significantly associated with injury rates (r = -0.181, *p* = 0.018) [8]. In FBI recruits, injuries have been associated with older age and decreased fitness levels [15]. Finally, in police officers, higher fitness levels increase the likelihood of completing recruit training [24]. Unfortunately, in all of these studies, training exposure within the recruit training program and beyond the recruit training program (i.e., what is done externally to the program) was not accounted for, thus limiting the clinical utility of these findings. Future research should be directed at clarifying the physical performance and psychological risk factors for injury in police training recruits to contribute to a broader understanding of the patterns of injury in this cohort and further guide injury prevention strategies.

### Limitations

Data linkage was piloted by two authors (NM and MM) to ensure the accuracy of the author who completed all data linkage (NM) with decisions on how to deal with missing data made via mutual agreement. The overseeing author (MM) has substantial experiencing leading studies involving data extraction and linkage [30–33]. With a single author completing all extraction and without all entries being independently cross-checked, minor coding errors may have occurred. Training days were used as the exposure variable in this study as this is what is recorded by the WA Police Force. However, more specific metrics (e.g., training session duration and rating of perceived exertion) would allow for more detailed statistical models. The Police Force injury database did not differentiate subsequent and recurrent injury, therefore we only included initial injuries within our regression model. The WA Police Force recruit injury database also had some missing demographic data (10.2%) and some injuries that were not accurately described which were subsequently assigned as ‘unspecified.’ This may have influenced the results, however given these data should be missing at random we do not think the influence is likely to be significant. These missing data are not specific to the WA Police Force records and are seen within other databases such as surgical [34] and elite sport settings [32]. Capturing as much information on the recruits’ demographic data is crucial for risk factor analyses [35] and to assist translation to clinical practice [36]. Future studies should consider the benefits of prospective data collection and monitoring as a strategy to reduce missing data. This would likely involve including the industry partner as a collaborator.

## Conclusion

This research has quantified the prevalence, incidence, severity and burden of injury to WA Police Force recruits when undergoing their recruit basic training program. Lower limb, shoulder, muscle/tendon and ligament/joint injuries present the largest injury burden to recruits during their training program. Being older than 30 years and being female were significant risk factors associated with injury. Further research to identify potentially modifiable risk factors and subsequent injury prevention interventions are needed.

## Supplementary Information

Below is the link to the electronic supplementary material.

Supplementary file1 (DOCX 67 KB)

## Data Availability

The data for this research study will not be publicly available as per agreement with the Western Australian Police Force. However, requests of the authors for access to the data or for analyses to be performed as a part of data synthesis will be considered and forwarded through to the Western Australian Police Force for approval.
