# Supplementary material for: Injury Profiles of Police Recruits Undergoing Basic Physical Training: A Prospective Cohort Study
Source: J Occup Rehabil. 2022 Aug 2;33(1):170–8. doi: 10.1007/s10926-022-10059-2 (PMC10025230; doi:10.1007/s10926-022-10059-2)

**Online resource A. Funnel plot’s depicting the injury burden in time-loss days per 1,000 recruit training days for: Top) injury area/ region, and Bottom) injury tissue pathology type.**


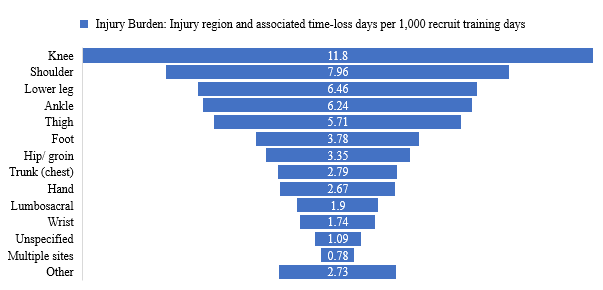

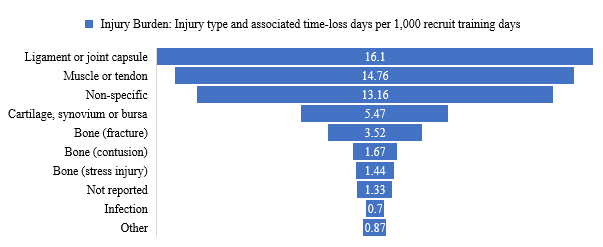


**Online resource B. Injury frequency for recruits by activity**

| **Injury activity** | **Frequency, n (%)** |
| --- | --- |
| Physical Training | 84 (31.8) |
| Own time | 52 (19.7) |
| Overuse | 21 (8.0) |
| Operational skills training | 20 (7.6) |
| Empty hand training | 21 (8.0) |
| Unspecified | 57 (21.6) |
| Not applicable | 8 (3.0) |
| Parade training | 1 (0.4) |

Legend: n=number, %= percentage.

**Online resource C. Cox regression time-to-event analysis assessing the effect of age, sex and training year on injury occurrence**

| **Variable** | **Effect size (B)** | **Standard Error** | **p-value** | **Hazard Ratio** | **95% CI of Hazard Ratio** |
| --- | --- | --- | --- | --- | --- |
| Training year | -0.0.15 | 0.065 | 0.811 | 0.985 | 0.867 to 1.118 |
| Sex (Male) | -0.550 | 0.133 | <0.001 | 0.577 | 0.444 to 0.749 |
| Age (30+) | 0.404 | 0.131 | 0.002 | 1.497 | 1.158 to 1.936 |

Legend: Effect size= B, CI= Confidence intervals

**Figure. Injury probability based on days of training: Cox-regression survival analysis**


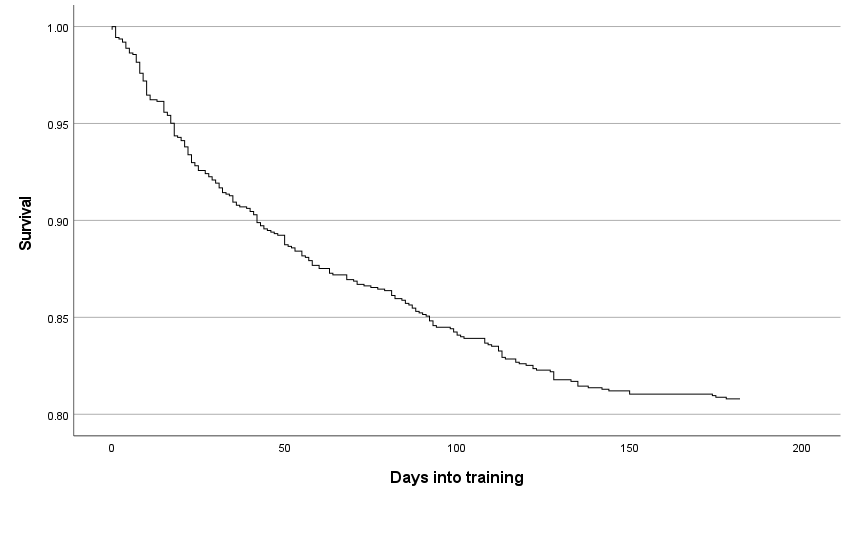

Supplement: Supplementary file 1 — Supplementary file1 (DOCX 67 KB) [file 10926_2022_10059_MOESM1_ESM.docx]
